# Psychometric properties of the Persian version of Depression Anxiety Stress Scale-21 Items (DASS-21) in a sample of health professionals: a cross-sectional study

**DOI:** 10.1186/s12913-022-07514-4

**Published:** 2022-01-26

**Authors:** Edris Kakemam, Elahe Navvabi, Ahmed Hassan Albelbeisi, Faeze Saeedikia, Amin Rouhi, Soheila Majidi

**Affiliations:** 1 Tabriz Health Services Management Research Center, Tabriz University of Medical Sciences, Tabriz, Iran; 2 Imam Hussein Hospital, Shahid Beheshti University of Medical sciences, Tehran, Iran; 3 Medical Services Directorate, Gaza Strip, Palestine; 4 Department of Health Services Management, School of Management and Medical Informatics, Tabriz University of Medical Sciences, Tabriz, Iran; 5 Deputy of Treatment, Hamadan University of Medical Sciences, Hamadan, Iran; 6 District Health Center of Hamadan City, Health Center of Hamadan City, Hamadan University of Medical Sciences, Hamadan, Iran

**Keywords:** DASS-21, Confirmatory factor analysis, Psychometric properties, Reliability, Validity, Iran

## Abstract

**Background:**

Although the Depression Anxiety Stress Scale-21 Items (DASS-21) has been used in different countries and translated into different languages, the Persian version of this scale has not been validated for healthcare professions in Iran. Therefore, the purpose of this study was to examine the psychometric properties of the Persian version of DASS-21 for nurses.

**Methods:**

This cross-sectional study was conducted among 1135 nurses working in public hospitals, who were selected through convenience sampling. DASS-21, which consists of 21 items and three dimensions (depression, anxiety, and stress), has been translated into Persian, and there is an online version available. A confirmatory factor analysis (CFA) was performed to examine the factor structure of this scale. Cronbach’s alpha coefficient was also measured to establish internal consistency. Besides, the intraclass correlation coefficient (ICC) was calculated to assess the test-retest reliability.

**Results:**

The Cronbach’s alpha coefficient was acceptable for anxiety (0.79), stress (0.91), and depression (0.93). An acceptable test-retest reliability (0.740-0.881, *P* < 0.01) was also reported for DASS-21 and its three dimensions. The results of CFA showed acceptable model fit (χ^2^/(df) = 1457/(186), *P* < 0.001), root mean square error of approximation (RMSEA = 0.078), Tucker-Lewis index (TLI = 0.906), comparative fit index (CFI = 0.917), and standardized root mean square residual (SRMR = 0.047). Fifty-seven nurses were included in the test-retest. The ICCs for all dimensions ranged from 0.75 to 0.86, indicating the acceptable test-retest reliability of the scale.

**Conclusion:**

The Persian version of DASS-21 showed good psychometric characteristics, and it was confirmed as a valid and reliable tool for evaluating depression, anxiety, and stress among Iranian nurses. However, further validation studies of the Persian DSASS-21 are needed among other healthcare professionals, including physicians, midwives, and allied health professionals.

## Background

Depression and anxiety are common problems around the world. The World Health Organization (WHO) estimated that more than 300 million people suffer from depression worldwide. Nearly the same number of people suffer from anxiety disorders, which is equivalent to almost 4-5% of the world’s population [1]. Depression is recognized as the most common mental health disorder globally and is expected to be the leading cause of mortality by 2030 [2, 3]. Economically disadvantaged developing countries have reported the highest prevalence of depression and anxiety symptoms [4]. Evidence suggests that these mental health problems are directly associated with social and physical problems, such as dysfunctional family relationships, high suicide rates, poor achievement, and use of illegal drugs [1, 5–7].

Today, it is clear that nurses have one of the most stressful health occupations, with more mental health complaints than any other health professionals [8–10]. Depression, anxiety, and stress are the most common mental health problems among nurses, which need to be considered in health research on Iranian nurses [11–13]. The high prevalence of depression, anxiety, and stress is associated with worse patient care safety and health care quality [14], higher occurrence of adverse events [15]. On the other hand, the COVID-19 pandemic has an important role in the well-being of healthcare professionals, especially nurses [16]. A systematic review and meta-analysis showed that almost one-third of nurses had depression, anxiety, and stress during the COVID-19 pandemic [17]. A study among Iranian nurses demonstrated that anxiety, stress, and depression scores significantly increased during the first wave of the COVID-19 pandemic compared with before the COVID-19 outbreak [18]. Additionally, a recent study focusing on comparison levels of depression, anxiety, and stress between nurses in the frontline and the second line of care delivery during the COVID-19 concluded that frontline line nurses had significantly higher levels of depression, anxiety, and stress [11].

The Depression Anxiety Stress Scale (DASS) is one of the screening instruments, designed to measure depression, anxiety, and stress. This validated questionnaire, which consists of 42 items, has been extensively used in different populations in various study backgrounds, given its applicability for the assessment of many negative mental health conditions [19]. DASS-21 is the new brief version of DASS with three subscales, each subscale includes seven items. Numerous studies have evaluated the psychometric properties of this scale in medical and non-medical populations to determine its validity and reliability [20–27]. The majority of these studies have reported good internal consistency and reliability [23–27].

Generally, DASS-21 is a low-cost, easy-to-use scale, which allows for a rapid assessment of depression, anxiety, and stress [28]. In this scale, it is presumed that depression, anxiety, and stress form a general distress construct, despite having distinguished features. The depression scale includes questions related to lack of interest, anhedonia, devaluation of life, self-deprecation, and inertia, while stress is associated with irritability, tension, nervousness, difficulty relaxing, and agitation [19].

DASS-21 has been translated into more than 40 languages, including Turkish, Greek, Malaysian, Korean, Vietnamese, and Brazilian Portuguese [23, 24, 27–29].

Although the DASS-21 has also been translated and validated among non-clinical Iranian samples [30], its Persian version has not been validated for the healthcare professions, especially nurses. Given the high prevalence of depression, anxiety, and other mental disorders in Iranian healthcare professions especially nurses and their different working environments, and conditions. DASS-21 may be a useful screening tool for identifying early-stage mental health symptoms. Further, considering the importance and impact of mental health disorders, a tool with good psychometric properties is important to accurately assess the level of depression, anxiety, and stress in a sample of health professionals. Therefore, the purpose of this study was to examine the psychometric properties of the Persian version of DASS-21 among a sample of health professionals.

## Methods

### Study design and setting

In this nationwide cross-sectional study, a convenience sampling method was applied. During the COVID-19 pandemic, nurses who worked in public hospitals between September 2020 and July 2021 were recruited in this study. A structured online questionnaire was distributed to gather information from the target population. This study is part of a larger study examining the relationship between burnout and depression, anxiety, and stress, based on self-reports of patient care quality and adverse events among nurses in Iran. One companion paper from this study assessed burnout and its relationship to self-reported quality of patient care and adverse events among nurses [31].

### Study sample

The inclusion criteria were as follows: nurses working full-time in public hospitals; nurses working in medical wards; and nurses with more than a year of working experience in the hospital. On the other hand, newly recruited nurses and part-time nurses were excluded.

### Sample size

The sample size was calculated based on an item-to-respondent ratio of 1:11 [32]. The minimum accepted sample size was 231 participants. However, 1135 nurses participated in the current study during the data collection period. To measure the test-retest reliability coefficient, a previous study showed that 52 participants are required [33]. Seventy participants were randomly selected to complete the questionnaires; also, 57 participants completed the questionnaire again after 3 weeks.

### Study instrument

In the current study, DASS-21 with 21 items and three subscales (7 items for each subscale) was used [34]. This instrument measures the prevalence of depression, anxiety, and stress signs and symptoms during the past weeks. The items of the scale are rated on a four-point Likert scale, with the score for each item ranging from 0 (“does not apply to me at all”) to 3 (“applies to me most of the time”). The subscale scores were calculated by summing the scores of the individual items, and the maximum sum for each subscale is 21; higher scores represent higher psychological distress. The original study reported the high reliability of DASS-21, Cronbach’s Alpha coefficients for depression, anxiety, and stress were reported as 0.91, 0.84, and 0.90, respectively [19]. The Persian version of DASS-21 has been previously translated and revised in an Iranian non-clinical setting [30]. This questionnaire includes seven items to assess the sociodemographic characteristics of individuals (i.e., gender, age, marital status, educational level, nursing experience, work unit, shift work, and work hours per week).

### Data collection

The study questionnaire was distributed online via Avalform (https://avalform.com), a viable survey tool widely used in Iran. At the beginning of the survey, a brief explanation was given regarding the purpose of the study and how to complete the questionnaire. After reading the explanations, if the participant responded positively to the question about willingness to participate in the study, he/she was allowed to enter the questionnaire; otherwise, he/she will not be able to access the survey questions. The answers to all questions were fixed, and the respondents could not proceed with the questionnaire unless all questions were completed; therefore, no data is missing. One answer to each question can be submitted via phone or computer account. The survey link was sent to the nurses via social media such as Telegram, WhatsApp and LinkedIn. Participating nurses were asked to send the link to other nurses in their departments and hospitals. To achieve a high response rate, the survey was sent out multiple times, and the survey link was available for about 12 weeks.

### Data analysis

We used SPSS V.20.0 and AMOS V.21.0 software of Windows for statistical analysis. Descriptive statistics were measured to describe the participants’ characteristics, each DASS-21 subscale, and individual items. Cronbach’s alpha coefficient was also determined to evaluate the internal consistency of DASS-21; a coefficient above 0.70 was reported, which is considered acceptable [35, 36]. Besides, corrected item-total correlations of the three subscales were calculated, which represent the correlation of each item with its subscale when the item is removed. The test-retest reliability method, based on the intraclass correlation coefficient (ICC) with a two-way random model, was used to evaluate reliability. An ICC above 0.7 indicates an acceptable test-retest reliability [37]. Moreover, the construct validity of DASS-21 was examined using a confirmatory factor analysis (CFA) to determine if the Persian version supports the three-factor construct of the original DASS-21. We did not use exploratory factor analysis (EFA) because as far as we knew, DASS-21 was developed using EFA [19]. In general, CFA is a robust method of statistical analysis for examining a predetermined factor structure or hypothetical theory; it describes how well each item evaluates the scale dimensionality. In many studies, CFA has been considered an important index for culturally adapted scales [38].

The goodness of fit was established based on the following fit indices: The Chi-square (χ^2^) goodness of fit should have a *P*-value > 0.05; the comparative fit index (CFI) should be ≥0.90 [39]; the Tucker–Lewis index (TLI) should be > 0.90 [40]; the root mean square error of approximation (RMSEA) should be ≥0.08 [40]; the goodness of fit index (GFI) and the adjusted goodness-of-fit index (AGFI) should be ≥0.85 [40], and the standardized root mean square residual (SRMR) should be ≤0.08 [40]. The correlations between the dimensions of DASS-21 were evaluated by measuring Pearson’s correlation coefficients.

## Results

### Characteristics of the study participants

The characteristics of the study respondents are presented in Table 1. A total of 1245 nurses completed this survey; however, 110 questionnaires were excluded, based on the inclusion and exclusion criteria. Finally, 1135 nurses were included in the analysis, with a mean age of 33.8 ± 7.8 years. The majority of the participants were female (75.7%), had shift work (77.4%), and had a bachelor’s degree (91.4%). The mean experience of the participants in nursing was 9.6 ± 7.3 years.

**Table 1:** Characteristics of the study participants (*N* = 1135)

Variable	Sub-category	N (%)
**Gender**	Male	276 (24.3)
	Female	859 (75.7)
**Age**	≤30	526 (46.3)
	31-40	371 (32.7)
	> 40	238 (21.0)
**Marital status**	Single	365 (32.2)
	Married	770 (67.8)
**Educational level**	Bachelor degree	1037 (91.4)
	Master or PhD	98 (8.6)
**Experience in nursing**	≤10	704 (62.0)
	> 10	431 (38.0)
**Current working unit**	Emergency	220 (19.4)
	Critical care units	282 (24.8)
	General wards	634 (55.8)
**Shift work**	Fixed	256 (22.6)
	Rotating	879 (77.4)
**Work hours per week**	≤42	334 (29.4)
	> 42	801 (70.6)

### Reliability

In Table 2 the results of the corrected item-total correlations and Cronbach’s alpha coefficients for the index subscales, in addition to the mean scores and standard deviations (SDs) for the items and the three subscales (depression, anxiety, and stress) was presented. The corrected item-total correlations for the DASS 21 were ranged from .59-.77 which could be considered acceptable, from the point of view that more than half of the retained elements have a score in the range of 0.30-0.70 [41]. The corrected item-total correlations were above 0.59 for the items of all subscales. The Cronbach’s alpha coefficient was 0.93 for the Persian version of DASS-21; it was 0.88 for depression, 0.88 for anxiety, and 0.89 for stress, indicating the acceptable internal consistency of the scale. In addition, the test-retest reliability of the Persian Version DASS-21 was acceptable, and the ICC of all domains was adequate which ranged from 0.75 to 0.86. Table 3 displays the test-retest reliability.

**Table 2:** Summary of reliability and mean scores and standard deviations for DASS-21 items and subscales (*n* = 1135)

Item (No. of items)	Corrected Item-Total Correlation	Cronbach’s Alpha if Item Deleted	Cronbach’s Alpha (95% CI^**a**^)	Mean (SD)
**DASS Depression**			**0.90 (0.89- 0.91)**	**9.58 (6.09)**
I could not seem to experience any positive feeling	0.70	0.89		1.33 (1.03)
I found it difficult to work up the initiative	0.52	0.91		1.18 (1.00)
I felt that I had nothing to look forward to	0.74	0.88		1.28 (1.15)
I felt down-hearted and blue	0.79	0.88		1.66 (1.11)
I was unable to become enthusiastic about anything	0.74	0.88		1.57 (1.05)
I felt I was not worth much as a person	0.73	0.89		1.13 (1.12)
I felt that life was meaningless	0.76	0.88		1.44 (1.17)
**DASS Anxiety**			**0.87 (0.86-0.88)**	**8.79 (5.59)**
I was aware of dryness of my mouth	0.59	0.86		1.52 (1.08)
I experienced breathing difficulty	0.61	0.86		1.14 (1.01)
I experienced trembling	0.67	0.85		1.06 (1.01)
I was worried about situations in which I might panic and make a fool of myself	0.62	0.86		1.36 (1.07)
I felt I was close to panic	0.72	0.85		1.29 (1.02)
I was aware of the action of my heart in the absence of physical exertion	0.67	0.85		1.25 (1.16)
I felt scared without any good reason	0.71	0.85		1.16 (1.05)
**DASS Stress**			**0.89 (0.88- 0.90)**	**1.82 (1.03)**
I found it hard to wind down	0.65	0.88		1.73 (0.89)
I tended to over-react to situations	0.62	0.88		1.47 (0.98)
I felt I was using a lot of nervous energy	0.67	0.87		2.13 (0.99)
I found myself getting agitated	0.72	0.87		1.41 (1.11)
I found it difficult to relax	0.77	0.86		1.42 (1.03)
I was intolerant of anything that kept from getting on with what I was doing	0.72	0.87		1.51 (1.01)
I felt that I was rather touchy	0.63	0.88		1.82 (1.03)

^**a**^ CI = Confidence Interval

**Table 3:** Test–retest reliability (*N* = 57)

DASS dimensions	ICC^**a**^ (95% CI^**b**^)	F Test	p
Depression	0.75 (0.42–0.88)	3.78	< 0.001
Anxiety	0.86 (0.70–0.94)	7.10	< 0.001
Stress	0.82 (0.59–0.92)	1.07	< 0.001

^a^ICC = Intraclass Correlation Coefficient. Two-way random

^b^CI = Confidence Interval

### Construct validity

**Fig. 1:**
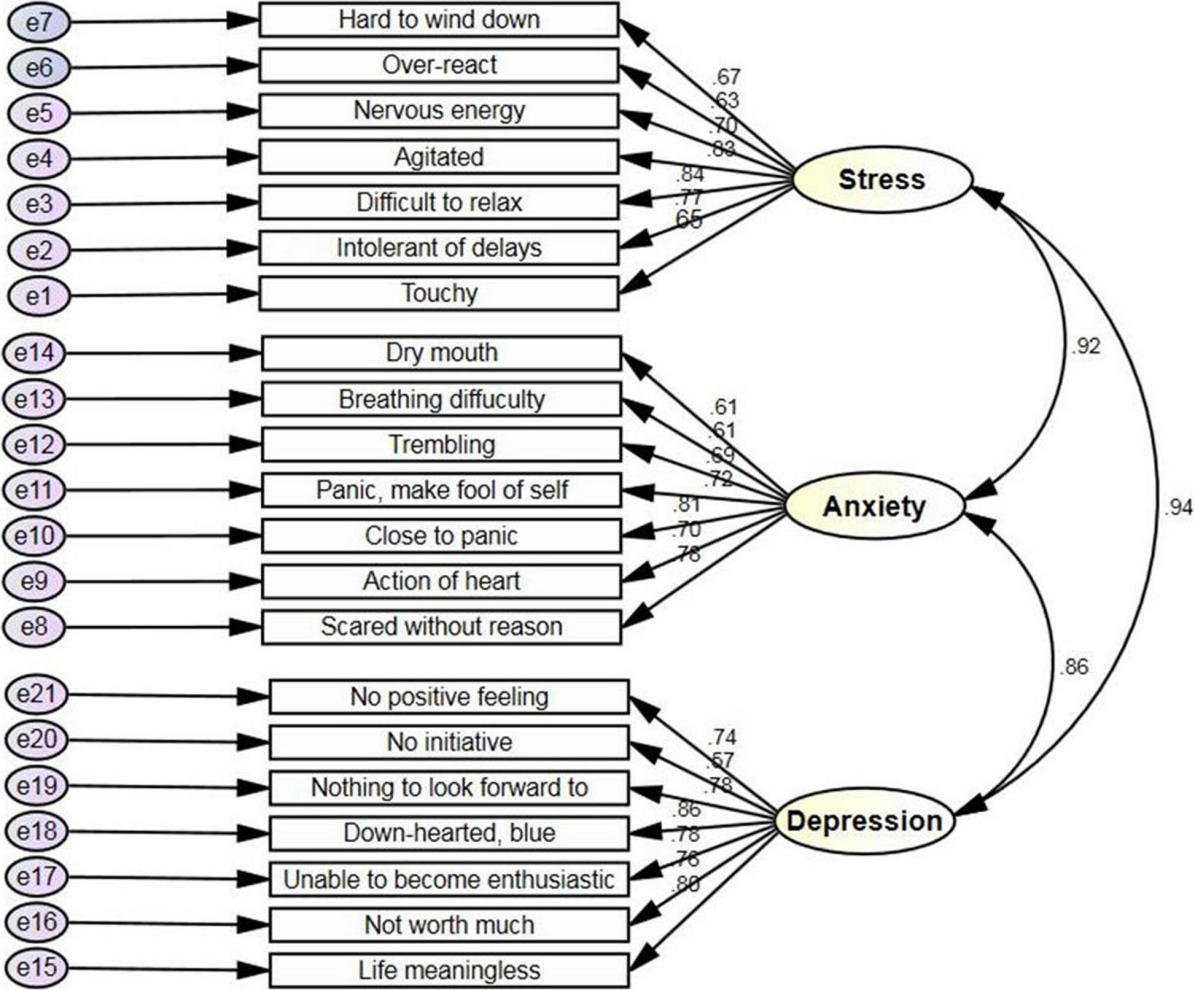
Confirmatory analysis model with factor loadings and correlations for the three IR- DASS-21 subscales

The analysis yielded a 21-item three-factor model that fit the data of the Persian of DAAS-21 (Fig. 1). The CFA revealed the acceptable fit indexes of the three-factor model (Table 4). The goodness-of-fit indices in the CFA showed RMSEA of 0.078, TLI of 0.91, CFI of 0.92, NFI of 0.91, SMRS of 0.047, and GFI of 0.88. Except for the AGFI, the fit indices were lower than the cutoff points in our study. Also, χ^2^/df was higher than the cutoff point (χ^2^/df = 7.83) [25, 38, 40, 42, 43]. The factor loadings ranged from 0.61 to 0.86 for all items, and the correlation coefficients between the subscales were between 0.86 and 0.94. Accordingly, the good fit of the three-factor model was established, suggesting the stability of this instrument.

**Table 4:** Confirmatory factor analysis fits for the three DASS-21 subscales (*n* = 1135)

Index	Index Criteria (***n*** > 250)	Fit Index in Iranian Sample (***n*** = 1135)
χ^2^ (df), *p*-value	*p* < 0.05	1457 (186), *p* < 0.001
Normed chi-square (χ2/df)	< 3	7.835
RMSEA (CI)	≤0.08	0.078
TLI	> 0.90	0.906
CFI	> 0.90	0.917
NFI	> 0.90	0.906
GFI	> 0.85	0.876
AGFI	> 0.85	0.847
SRMR	≤0.08	0.047

*RMSEA* Root mean square error of approximation, *CI* confidence interval, *TLI* Tucker-Lewis index, *CFI* Comparative fit index, *NFI* Normed fit index, *GFI* Goodness-of-fit index, *AGFI* Adjusted goodness-of-fit index, *SRMR* Standardized root mean square residual

### Correlations between the subscales of the Persian version of DASS-21

Table 5 presents the correlations (r) between the subscales of the Persian DASS-21. The subscales of the Persian DASS-21 were found to be significantly correlated. The coefficients for the correlations of subscales were as follows: 0.77 for depression and anxiety; 0.83 for depression and stress; and 0.82 for anxiety and stress.

**Table 5:** Pearson correlations among the subscales of the IR- DASS-21 (*n* = 1135)

	DASS Depression	DASS Anxiety	DASS Stress
DASS Depression	–	0.77**	0.83**
DASS Anxiety		–	0.82**
DASS Stress			–

**p < 0.01

## Discussion

Although DASS-21 has been used in different countries and translated into different languages, the Persian version of this scale has not been validated in medical populations in Iran. The current study investigated the psychometric properties of the Persian version of DASS-21 for a sample of health professionals. This is the first study, to the best of our knowledge, to examine the factor structure of the Persian DASS-21 for healthcare professionals. The present findings supported the three-factor structure of DASS-21, as reported in previous studies [19, 23, 25, 26, 29, 30, 36].

The main contribution of the current study was the measurement of the psychometric properties of the Persian version of DASS-21 among nurses as important health professionals in any healthcare system, contributing significantly to the delivery and quality of healthcare services [44–46]. In Iran, there is a shortage of nurses of around 130,000, and there is and only 1.3 nurses per 1000 people [47], compared with the Organization for Economic Co-operation and Development (OECD) average of 7.4 per 1000 people [48]. A recent systematic review study identified he most important causes for the shortage of nurses in Iran. they including unwillingness to enter and stay in nursing, job abandonment and trying other jobs, low social status, insufficient support in the workplace, immigration to other countries, poor policies and programs for recruitment of workforce, the negative impression of nursing as a woman’s career, Insufficient salaries compared to other professions [47]. On the other hand, Iranian nurses face many challenges such as job dissatisfaction [49], high job stress [15], low quality of work life [50], high job burnout [31] and high anxiety and depression [51].

As frontline health service providers, nurses may face different challenges, such as the risk of infection, lack of protective supplies, risk of infection by relatives, and lack of essential medications; these challenges may increase the risk of insomnia, frustration, fear, stress, anxiety, and depression among nurses [52–54]. During the current COVID-19 pandemic, many studies around the world and in Iran have indicated the urgent need to evaluate psychological disorders in the general population, as well as healthcare professionals, who are at the highest risk of psychological and mental health problems [55–57].

In the present study, the internal consistency reliability and construct validity of the Persian DASS-21 were examined. This validated questionnaire can be used as a robust tool to measure depression, anxiety, and stress among Iranian healthcare professionals, particularly nurses. The present results revealed the acceptable internal consistency reliability of the scale, with a Cronbach’s alpha coefficient of 0.88 for depression, 0.88 for anxiety, and 0.89 for stress; the overall Cronbach’s alpha coefficient was 0.93. The present results are consistent with previous validation studies of DASS-21 conducted in different countries in both medical and non-medical settings, which revealed that Cronbach’s alpha was above the agreed cutoff point (0.70) [22–26, 29, 30, 58]. Psychometric studies of the DASS tool for healthcare professions in Turkey and Greece showed that Cronbach’s alpha coefficient for three dimensions varies between 0.81 and 0.85 [23, 24]. Moreover, a study in China among hospital workers reported a Cronbach’s alpha coefficient of 0.95 for the total DASS-21 scale [22].

The construct validity of the Persian DASS-21 was satisfactory based on the CFA, and all fit indexes of the model were acceptable. Generally, structural validity refers “to the extent to which the structure of a multi-item scale reflects the hypothesized dimensionality of the construct being measure” [59]. The indexes of the CFA model showed that the three-dimension structure of DASS-21 was a satisfactory fit for the data, and the results were in line with previous validation research [22, 24, 60]. The findings of the present study showed that the Persian DASS-21 is suitable for future research in the Iranian community.

In the present study, the RMSEA was calculated to be 0.078, and χ^2^/df *P*-value was < 0.001. Based on the criteria for CFA, the goodness-of-fit indices of CFA indicated the acceptable fit of the model with the original construct [40]. Some previous studies have reported acceptable RMSEA, including a study conducted in Turkey among health control and clinical samples (RMSEA = 0.065) [24] and reported a χ^2^/df P-value of > 0.05. According to the criteria for CFA, these goodness-of-fit indices suggest the acceptable fit of the model with the original construct [40].

Besides, some studies have reported acceptable RMSEA, including a study conducted in Turkey among health control and clinical samples (RMSEA = 0.065) [24] and a study in Chinese hospital workers (RMSEA = 0.075) [22]. Moreover, the CFI measured in our study (0.917) was above the cutoff point, indicating a satisfactory model fit. It was lower than the values reported in studies performed in Australia (CFI = 0.944) [61] and Greece (CFI = 0.95) [23], while it was higher than the value reported in Turkey (CFI = 0.905) [24].

### Limitations

There are some limitations to the present study, Due to coronavirus concerns, data was collected online and using a convenient sampling method, which could result in selection bias. Also, only nurses with access to the Internet could complete the questionnaire. Besides, other healthcare professionals, such as physicians, laboratory technicians, and paramedics, were not included in this study. Nevertheless, the present study established the validity and reliability of the Persian version of DASS-21, which can be applied in future research to measure depression, anxiety, and stress in different healthcare professionals in the Iranian healthcare system.

## Conclusion

The Persian version of DASS-21 showed good psychometric characteristics. This tool was found to be a valid and reliable questionnaire to assess depression, anxiety, and stress among Iranian nurses. However, further validation studies of this scale are needed among other healthcare professionals, including physicians, midwives, and allied health professionals.

## Data Availability

The datasets used and/or analyzed during the current study are available from the corresponding author on reasonable request.

## Abbreviations

AGFI: Adjusted goodness-of-fit index
CFA: Confirmatory factor analysis
CI: Confidence Interval
DASS-21: Depression Anxiety Stress Scale-21 Items
GFI: Goodness of fit index
ICC: Intraclass correlation coefficient
RMSEA: Root mean square error of approximation
SD: Standard deviations
SRMR: Standardized root mean square residual
TLI: Tucker-Lewis index
WHO: World Health Organization
